# Intramyocardial fatty infiltration lesion in sporadic inclusion body myositis: a case report

**DOI:** 10.1007/s10554-024-03271-z

**Published:** 2024-10-28

**Authors:** Betim Redzepi, Marie Théaudin, Samir Bengueddache, Sofia Petropoulou-Natsou, Ambra Masi, David Rodrigues, Georgios Tzimas, Juerg Schwitter, Panagiotis Antiochos

**Affiliations:** 1 Cardiovascular Department of Cardiology, Division of Cardiology, Lausanne University Hospital, CHUV, Rue du Bugnon 46, Lausanne, 1005 Switzerland; 2 Cardiac MR Center of the University Hospital Lausanne, CHUV, Lausanne, Switzerland; 3 Department of Neurology, Lausanne University Hospital, CHUV, Lausanne, Switzerland; 4 Faculty of Biology and Medicine, University of Lausanne, UniL, Lausanne, Switzerland; 5 Department of Diagnostic and Interventional Radiology, Lausanne University Hospital, CHUV, Lausanne, Switzerland

**Keywords:** Sporadic inclusion body myositis, Intramyocardial fatty infiltration, Cardiac magnetic resonance, Case report

## Abstract

Sporadic inclusion body myositis (sIBM), the most common inflammatory muscle disorder in adults over 50 years, is often misdiagnosed due to its gradual onset and its common but unspecific muscle weakness in older adults. Diagnosis relies on clinical, radiological, and pathological features. Cardiac involvement is rare, prompting this case description and a comprehensive literature analysis. A 73-year-old woman diagnosed with sIBM in 2021 through muscle biopsy had been experiencing muscular symptoms since 2015. Her condition progressively worsened, affecting daily activities. Annual follow-ups revealed a moderate obstructive syndrome on respiratory testing, prompting a cardiac evaluation. Cardiac magnetic resonance (CMR) imaging identified intramyocardial lesions consistent with fatty infiltration, highlighting the interest of advanced imaging in sIBM management. Cardiac involvement in sIBM is presumed rare compared to other idiopathic inflammatory myopathies, though the exact frequency remains unclear. Early identification of heart alterations by CMR in sIBM can be prognostically valuable, guiding follow-up and interventions. However, literature on this subject is limited to small cohort studies and case reports describing complications. Given the slow progression of sIBM and the limited efficacy of current treatments, the discovery of myocardial lesions could warrant closer cardiological monitoring. Larger cohort studies are needed to explore potential new therapeutic approaches. Our case underscores the importance of CMR in detecting subtle cardiac manifestations in sIBM and illustrates the potential prognostic value of cardiac assessment in the management of sIBM.

## Introduction

Sporadic inclusion body myositis (sIBM) is the most common and disabling inflammatory skeletal muscle disorder among adults over 50 years of age [1]. It occurs in 15 to 70 per million [2]. The high prevalence of muscle weakness in older adults from various etiologies, combined with the gradual and subtle onset of weakness in sIBM, makes this rare disease frequently misdiagnosed [3, 4]. Diagnostic criteria of sIBM include clinical, radiological, and pathological features [5, 6]. In any patient developing slowly progressive muscle weakness, particularly involving the quadriceps muscles and wrist/finger flexors, even without an elevated serum creatine kinase, referral to a neurologist and muscle biopsy is recommended [7–9]. Current immunosuppressive and immunomodulatory treatments have shown limited effectiveness [10]. 

In this disorder, data on cardiac involvement are scarce and limited to case reports and small case series, probably because cardiac involvement in sIBM is considered uncommon compared to other idiopathic inflammatory myopathies, though its precise frequency is unknown.

Therefore, after describing a case with sIBM and intramyocardial lesions consistent with fatty infiltration, we conducted a comprehensive analysis of the existing literature on cardiac involvement in sIBM and identified gaps for future research.

## Case description

We report the case of a 73-year-old woman who was followed in the neurology clinic for a sIBM. She had been symptomatic since 2015 and was diagnosed with sIBM based on her clinical symptoms and a muscle biopsy of the left quadriceps on August 3, 2021. She presented with a typical slightly asymmetrical motor deficit in the deep finger flexors and anterior tibialis but normal quadriceps. At her last visit in the neurology department in 2023, she complained of a slowly progressive increase of the muscle weakness in all four limbs, leading to daily life activity difficulties together with leg fatigability for prolonged walks. She did not experience any dyspnea and reported no palpitations or syncopal episodes.

**Fig. 1:**
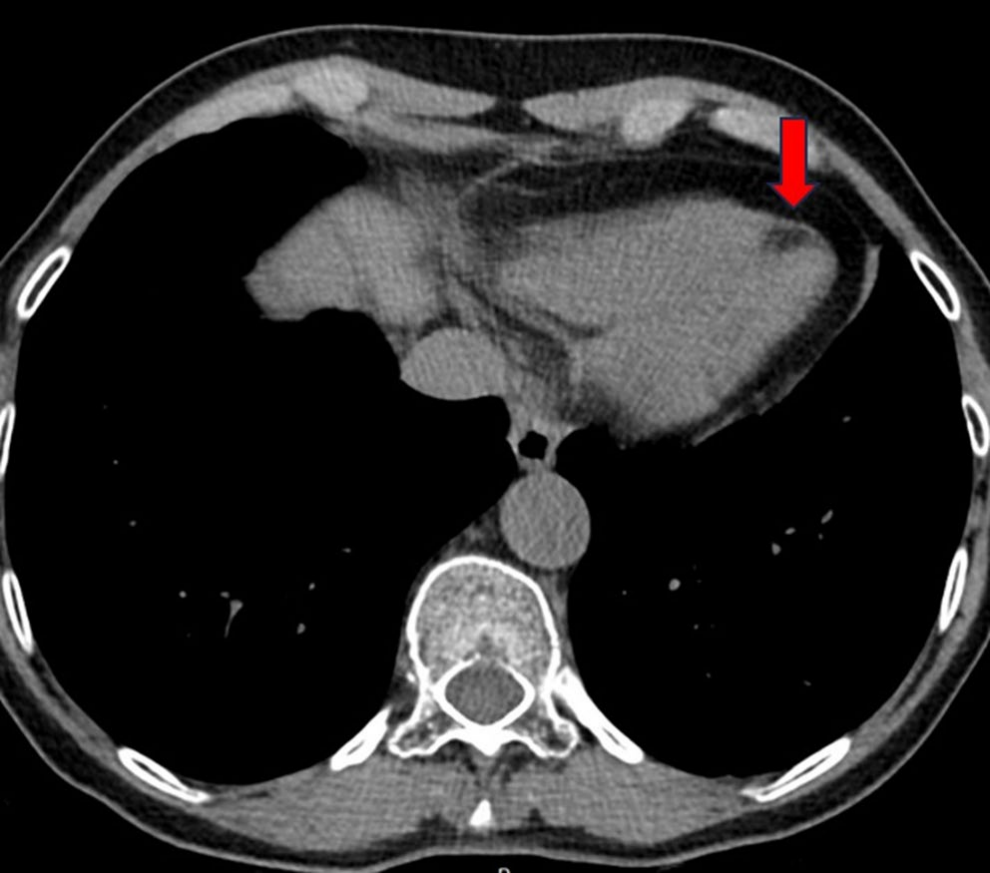
Thoracic CT scan showing a hypodense lesion with negative attenuation values (-77 Hounsfield units) located at the apex of the left ventricle (red arrow)

**Fig. 2:**
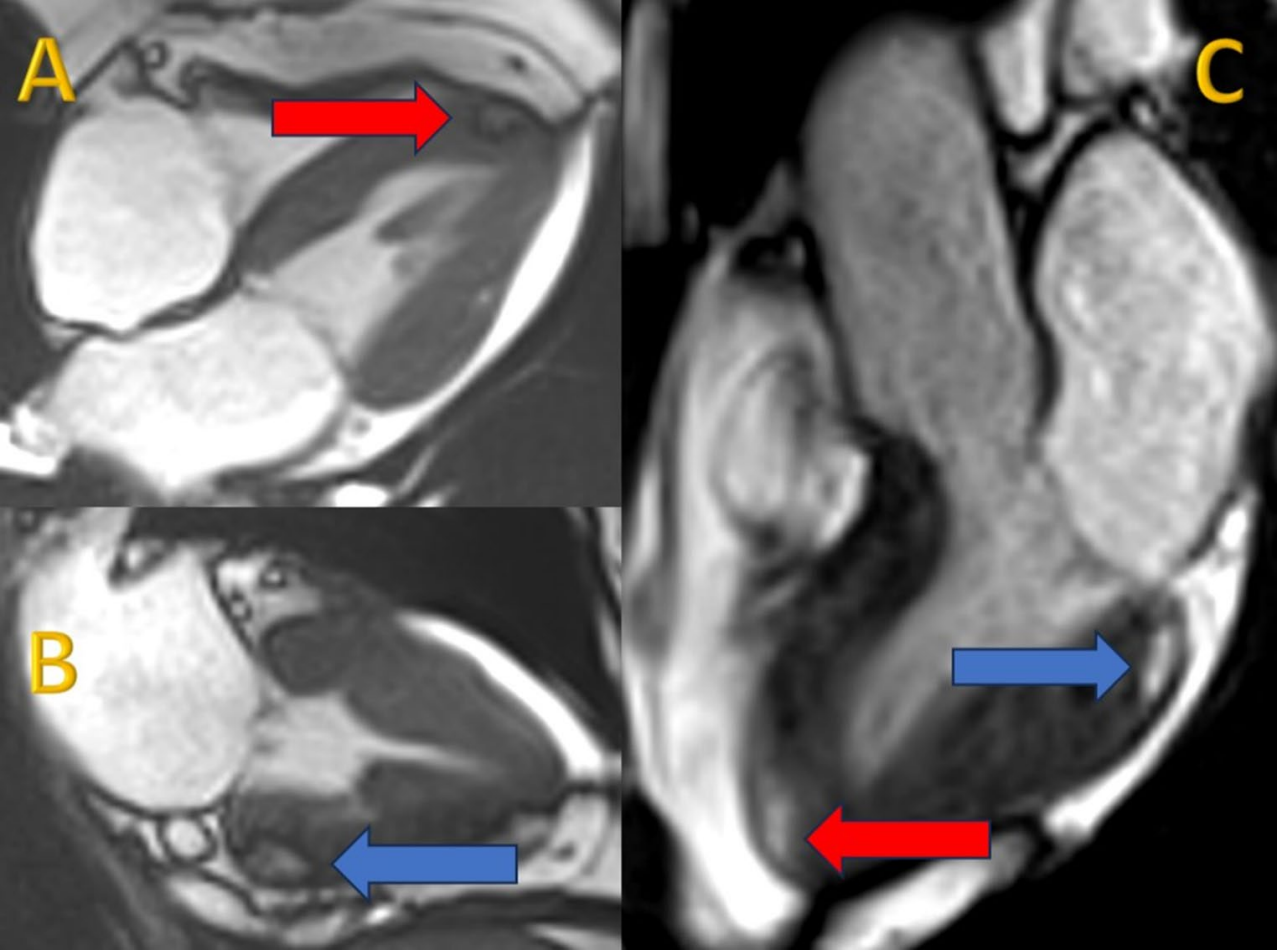
Steady-state free precession (SSFP) cine sequence four-chamber view (**A**) showing the apical lesion (red arrow), two-chamber view (**B**) showing the basal inferior lesion (blue arrow) and three-chamber view (**C**) showing the apical lesion (red arrow) and the basal inferolateral lesion (blue arrow). The dark rim surrounding the lesions is a typical artifact caused by fat (chemical shift artifact)

**Fig. 3:**
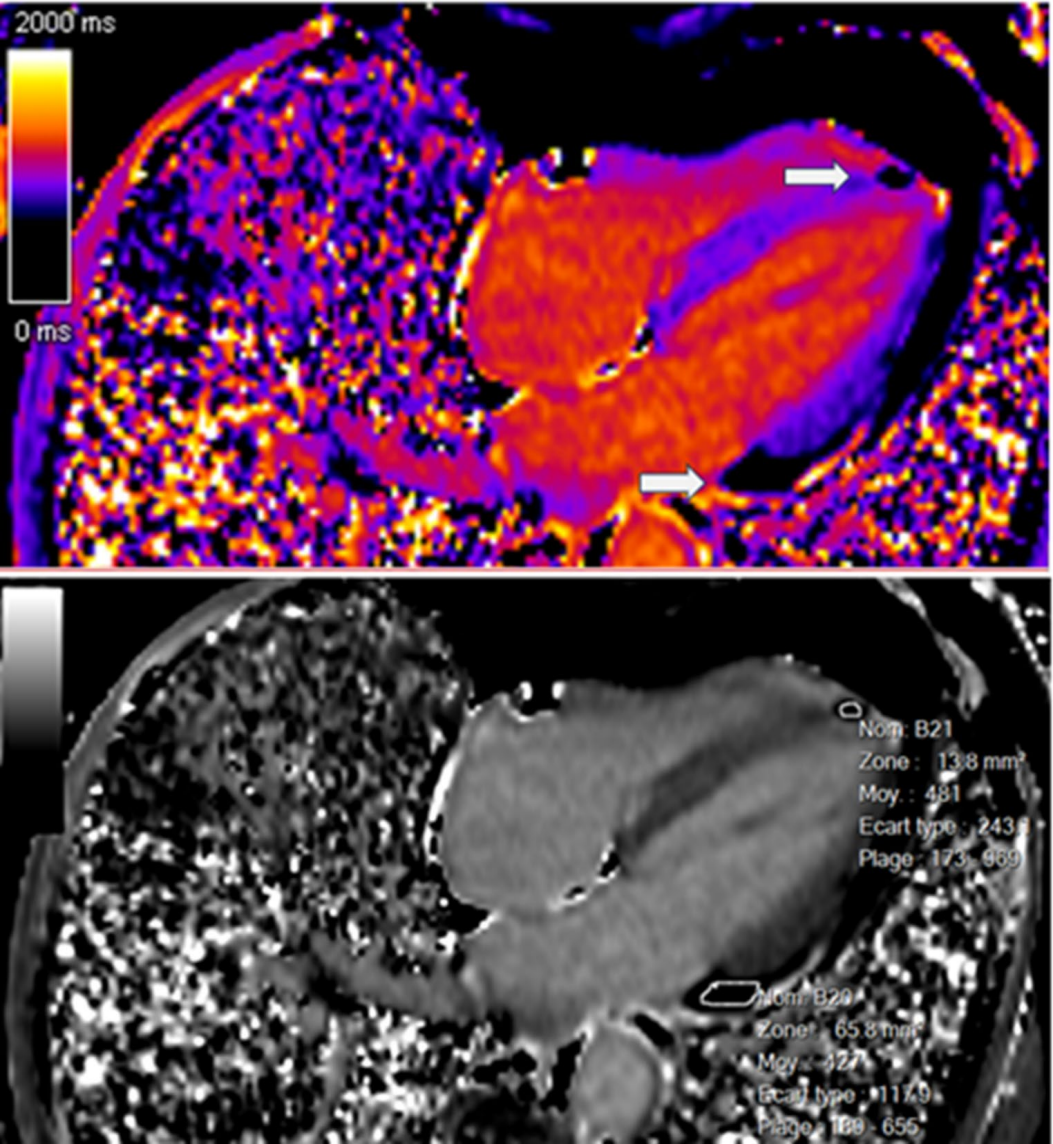
Lesions (white arrows) visualized in a four-chamber native (precontrast) T1-mapping view and characterized by a reduced native T1 relaxation time (427-481milliseconds)

**Fig. 4:**
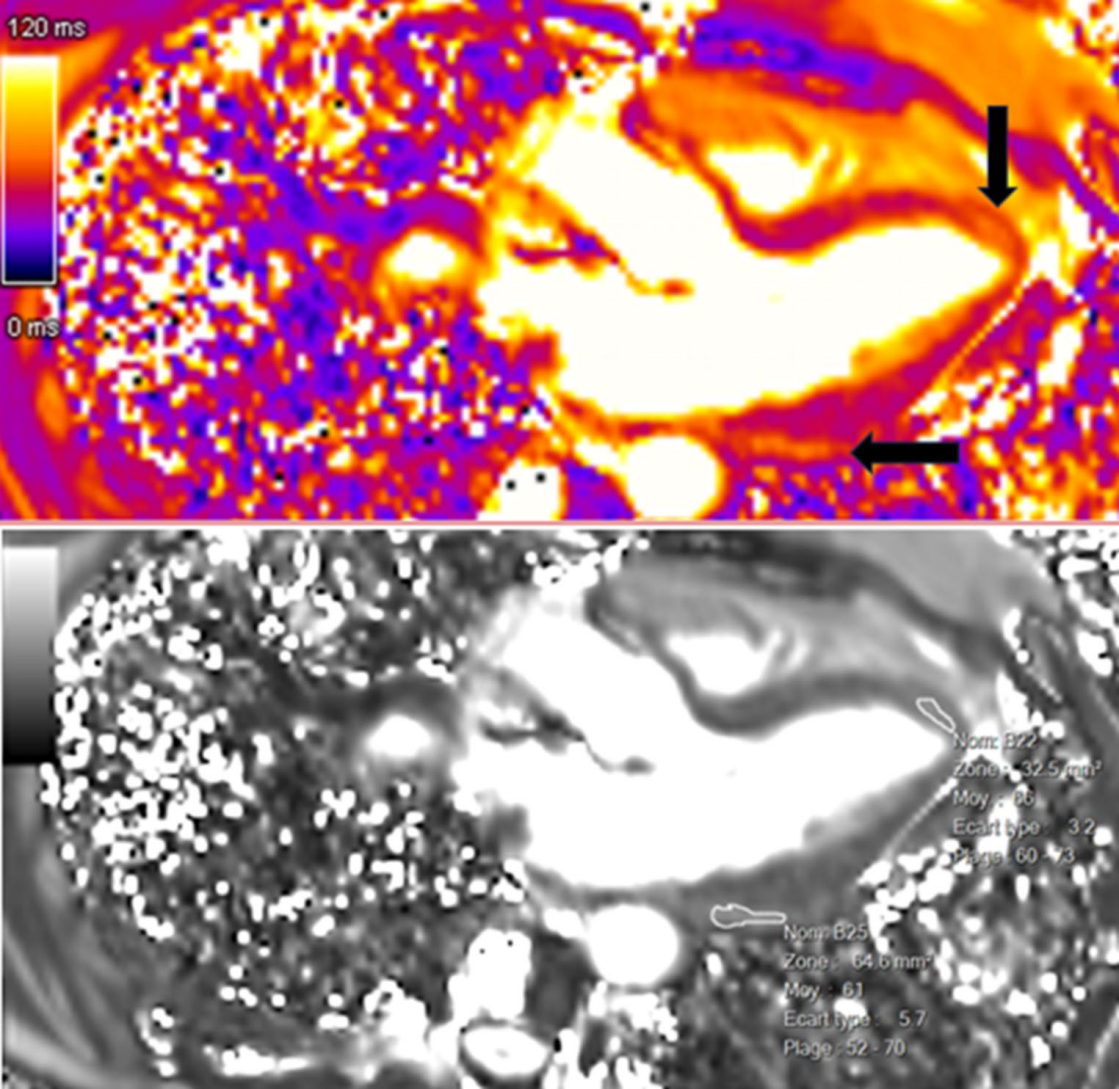
Lesions (black arrows) visualized in a three-chamber native (precontrast) T2-mapping view and characterized by an increased native T2 relaxation time (61-66milliseconds)

**Fig. 5:**
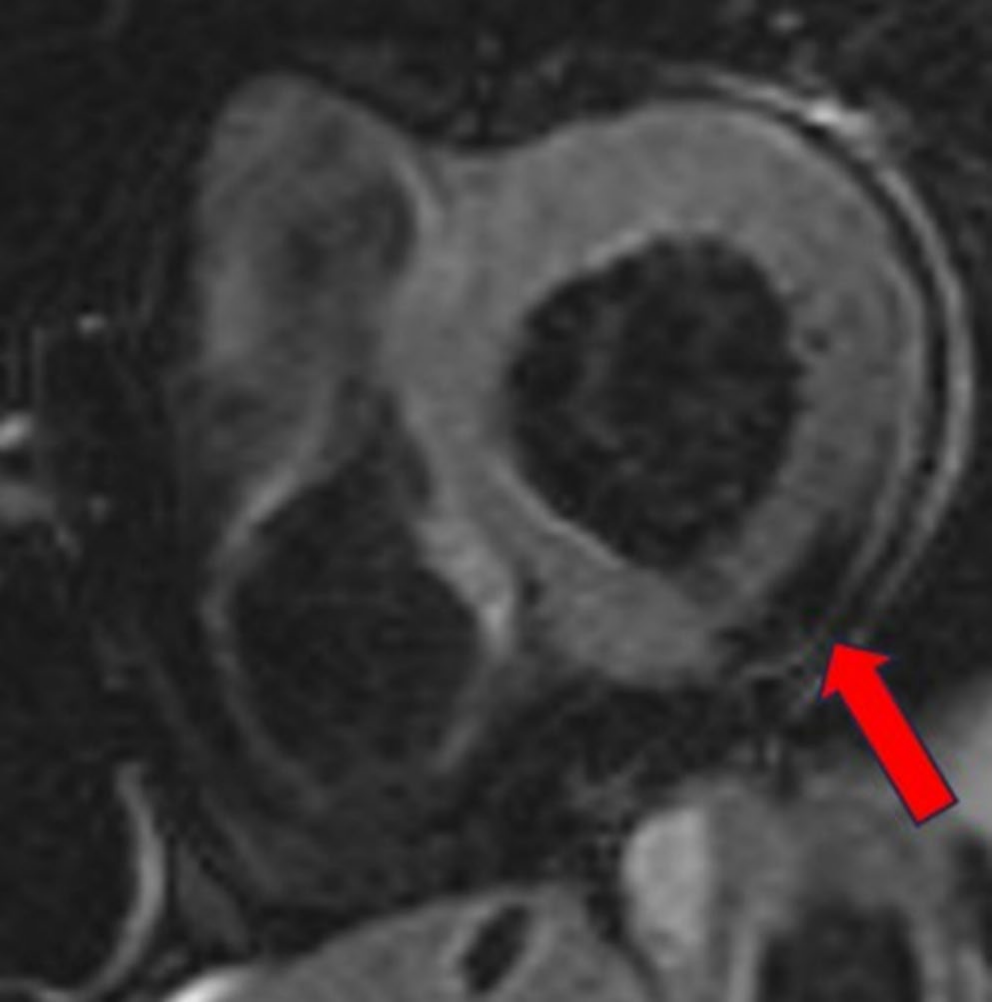
Hypointense signal in basal inferior lesion (red arrow) on spectral attenuated inversion recovery (SPAIR) sequence in a basal short-axis view

In the context of her muscular disease, she had annual pulmonary and ear, nose and throat follow-ups. When the functional respiratory test showed a non-reversible moderate obstructive syndrome, the patient had a thoracic CT scan, which was normal except for an incidental hypodense lesion at the apex of the left ventricle (Fig. 1). The patient was thus referred to the cardiology division. Echocardiography was normal except for a dyskinetic aneurysmal movement at the apex of the left ventricle without pericardial effusion. A cardiac magnetic resonance (CMR) examination (Siemens Healthineers Magnetom Sola, 1.5 Tesla) with multiparametric techniques, including native T1 and T2 mapping, extracellular volume (ECV), spectral attenuated inversion recovery (SPAIR) for fat suppression and late gadolinium enhancement (LGE) showed two intramyocardial lesions. The first one in the septal apex, measured 15 × 5 millimeters and the other in the inferior basal segment extending into the inferolateral and anterolateral segments, 12 × 7 millimeters (Fig. 2A and C), with the common following characteristics: decreased native T1 values of 427–481 ms (normal values between 970 and 1116 ms) [11] (Fig. 3), elevated native T2 values of 61–66 ms (normal values between 51 and 57 ms) [12] (Fig. 4). The hypointense signal in the fat-suppressing SPAIR sequences (Fig. 5) confirmed the presence of myocardial fatty infiltration. Intramyocardial scarring was excluded by the absence of LGE and normal ECV. Due to the absence of cardiovascular symptoms, no cardiac biopsy was performed.

## Discussion

sIBM is a progressively worsening inflammatory muscle disorder marked by muscle weakness and atrophy, particularly in the quadriceps and finger flexors. While the exact cause remains unclear, it is believed to be a complex condition with various contributing factors, including immune-mediated muscle damage and degenerative changes [13]. Magnetic resonance imaging of the thigh in patients with sIBM typically shows edema, atrophy and fatty replacement of the anterior compartment with relative sparing of the medial and posterior compartments [14, 15]. On histopathology, the condition commonly exhibits inflammatory infiltrates in muscle tissue, primarily composed of cytotoxic T cells and macrophages. Autoantibodies such as anti-cN1A have been detected in some sIBM patients, suggesting a possible autoimmune aspect [16, 17]. Additionally, histopathological changes detectable by light microscopy often reveal rimmed vacuoles, cytoplasmic protein aggregates, and mitochondrial abnormalities, indicating a degenerative component that may be related to protein misfolding and impaired autophagy [13, 16, 18]. 

Compared to other idiopathic inflammatory myopathies, such as dermatomyositis and polymyositis with a cardiac involvement of between 6 and 75% with a variability depending on a definition of heart involvement and the detection method used, cardiac involvement in sIBM is considered rare, although the exact frequency also remaining unclear [3, 19]. Cardiac involvement has been rarely reported with, mainly, dilated cardiomyopathy and complex ventricular arrhythmias [20, 21]. There was no description of focal fatty lesions in any of these cases. However, CMR assessment was not done in all cases. In 2010, a cohort study included 51 sIBM patients and looked for heart involvement with electrocardiographic and echocardiographic data [22]. No evidence of cardiac involvement was found, leading the authors to conclude that routine comprehensive cardiac evaluation in sIBM patients without cardiac symptoms should not be recommended. However, CMR was not performed in that study.

Another recent study on 20 sIBM patients without cardiovascular symptoms showed LGE patterns consistent with myocardial fibrosis in 7 (35%) patients [23]. Although the authors concluded that it was a nonspecific finding, one could hypothesize that without conducting a multiparametric CMR study, subclinical cardiac involvement might not have been detected. Indeed, in sIBM patients, patchy intramural LGE was located in the inferior and inferolateral segments.

Our case report is the first, to our knowledge, that found fatty intramyocardial infiltrations detected by specific multiparametric analysis in the absence of scar or fibrosis in sIBM. Fatty infiltration of the heart observed on CMR can indicate various conditions that should be considered in the differential diagnosis, including arrhythmogenic right ventricular cardiomyopathy (ARVC), cardiac sarcoidosis, and Neurofibromatosis type 1 (Recklinghausen Disease). Specifically regarding ARVC, recent studies suggest that a “bite-like” fibrofatty replacement pattern in the left ventricle is highly specific for ARVC with left ventricular involvement when assessed within the appropriate clinical context [24]. In the absence of an alternative explanation, such as sIBM, the presence of this feature in a patient with suspected ARVC should strongly prompt suspicion of the disease and warrant a comprehensive diagnostic evaluation, including resting ECG, Holter monitoring, and genetic testing. These findings must always be correlated with both clinical presentation and CMR results for accurate diagnosis.

Early identification of morphologic and functional alterations in the heart can probably be of prognostic value. Though the exact percentage can vary slightly depending on the study and cohort characteristics, cardiovascular causes are responsible for approximately 20–30% of deaths in patients with sIBM due to a combination of factors including chronic muscle inflammation, reduced physical activity and associated comorbidities such as hypertension and diabetes. Therefore, monitoring disease and in particular cardiac involvement may guide the initiation and monitoring of various interventions. In this context, CMR appears to be the imaging method of choice. Additionally, this disease is slowly progressive, and current immunosuppressive and immunomodulatory treatments have shown limited effectiveness [10, 25]. Therefore, scientific societies recommend the implementation of pharmacological treatment in cases of severe inflammation disproportionate to fibrosis on muscle biopsy and/or magnetic resonance imaging or in those patients with other autoimmune diseases, which are more commonly associated with cardiac involvement. Hence, the discovery of myocardial lesions in sIBM should prompt closer cardiological follow-up. In order to better understand if these fatty lesions could be frequently associated with sIBM, large cohort studies should be performed, which may secondarily drive the development of new therapeutic modalities.

## Conclusion

In conclusion, while previous studies questioned the need for routine cardiac assessments in sIBM without evident symptoms, our paper suggests that subtle cardiac manifestations may be missed without specific imaging modalities. Larger cohort studies including multiparametric CMR may shed a light on this rare disease and may demonstrate the potential prognostic value of cardiac assessments in the management of sIBM.

## Data Availability

No datasets were generated or analysed during the current study.

## References

[1] Weihl CC, Mammen AL (2017) Sporadic inclusion body myositis - a myodegenerative disease or an inflammatory myopathy. Neuropathol Appl Neurobiol 43(1):82–91. 10.1111/nan.12384 28111778 10.1111/nan.12384

[2] McLeish E, Slater N, Sooda A et al (2022) Inclusion body myositis: the interplay between ageing, muscle degeneration and autoimmunity. Best Pract Res Clin Rheumatol 36(2):101761. 10.1016/j.berh.2022.101761 35760741 10.1016/j.berh.2022.101761

[3] Shelly S, Mielke MM, Mandrekar J et al (2021) Epidemiology and natural history of inclusion body myositis: a 40-Year Population-based study. Neurology 96(21):e2653–e2661. 10.1212/WNL.0000000000012004 33879596 10.1212/WNL.0000000000012004 PMC8205447

[4] Needham M, Corbett A, Day T, Christiansen F, Fabian V, Mastaglia FL (2008) Prevalence of sporadic inclusion body myositis and factors contributing to delayed diagnosis. J Clin Neurosci 15(12):1350–1353. 10.1016/j.jocn.2008.01.011 18815046 10.1016/j.jocn.2008.01.011

[5] Lloyd TE, Mammen AL, Amato AA, Weiss MD, Needham M, Greenberg SA (2014) Evaluation and construction of diagnostic criteria for inclusion body myositis. Neurology 83(5):426–433. 10.1212/WNL.0000000000000642 24975859 10.1212/WNL.0000000000000642 PMC4132572

[6] Jones K, Pitceathly RDS, Rose MR et al (2016) Interventions for dysphagia in long-term, progressive muscle disease. Cochrane Database Syst Rev 2(2):CD004303. 10.1002/14651858.CD004303.pub4 26859621 10.1002/14651858.CD004303.pub4 PMC8759487

[7] Kley RA, Vorgerd M, Tarnopolsky MA (2007) Creatine for treating muscle disorders. Cochrane Database Syst Rev 1CD004760. 10.1002/14651858.CD004760.pub2 10.1002/14651858.CD004760.pub2 17253521

[8] Brady S, Squier W, Sewry C, Hanna M, Hilton-Jones D, Holton JL (2014) A retrospective cohort study identifying the principal pathological features useful in the diagnosis of inclusion body myositis. BMJ Open 4(4):e004552. 10.1136/bmjopen-2013-004552 24776709 10.1136/bmjopen-2013-004552 PMC4010816

[9] de Camargo LV, de Carvalho MS, Shinjo SK, de Oliveira ASB, Zanoteli E (2018) Clinical, histological, and immunohistochemical findings in inclusion body myositis. Biomed Res Int 2018:5069042. 10.1155/2018/5069042 29780824 10.1155/2018/5069042 PMC5893008

[10] Needham M, Mastaglia FL (2016) Sporadic inclusion body myositis: a review of recent clinical advances and current approaches to diagnosis and treatment. Clin Neurophysiol 127(3):1764–1773. 10.1016/j.clinph.2015.12.011 26778717 10.1016/j.clinph.2015.12.011

[11] Rosmini S, Bulluck H, Captur G et al (2018) Myocardial native T1 and extracellular volume with healthy ageing and gender. Eur Heart J Cardiovasc Imaging 19(6):615–621. 10.1093/ehjci/jey034 29617988 10.1093/ehjci/jey034 PMC5963299

[12] Hashemi S, van Heeswijk R, Schwitter J, Hullin R, Stube M, Schwitter J (2018) Comparison of three different Cardiac T2-Mapping techniques at 1.5 Tesla. Biomed J Sci &Tech Res 3(2):3143–3150. 10.26717/BJSTR.2018.03.000876

[13] Greenberg SA (2019) Inclusion body myositis: clinical features and pathogenesis. Nat Rev Rheumatol 15(5):257–272. 10.1038/s41584-019-0186-x 30837708 10.1038/s41584-019-0186-x

[14] Zubair AS, Salam S, Dimachkie MM, Machado PM, Roy B (2023) Imaging biomarkers in the idiopathic inflammatory myopathies. Front Neurol 14:1146015. 10.3389/fneur.2023.1146015 37181575 10.3389/fneur.2023.1146015 PMC10166883

[15] Tasca G, Monforte M, De Fino C, Kley RA, Ricci E, Mirabella M (2015) Magnetic resonance imaging pattern recognition in sporadic inclusion-body myositis. Muscle Nerve 52(6):956–962. 10.1002/mus.24661 25808807 10.1002/mus.24661

[16] Dimachkie MM (2011) Idiopathic inflammatory myopathies. J Neuroimmunol 231(1–2):32–42. 10.1016/j.jneuroim.2010.10.013 21093064 10.1016/j.jneuroim.2010.10.013

[17] Jiang R, Roy B, Wu Q et al (2023) The plasma cell infiltrate populating the muscle tissue of patients with inclusion body myositis features distinct B cell receptor Repertoire Properties. Immunohorizons 7(5):310–322. 10.4049/immunohorizons.2200078 37171806 10.4049/immunohorizons.2200078 PMC10579972

[18] Lindgren U, Roos S, Hedberg Oldfors C, Moslemi AR, Lindberg C, Oldfors A (2015) Mitochondrial pathology in inclusion body myositis. Neuromuscul Disord 25(4):281–288. 10.1016/j.nmd.2014.12.010 25638290 10.1016/j.nmd.2014.12.010

[19] Zhong Y, Bai W, Xie Q, Sun J, Tang H, Rao L (2018) Cardiac function in patients with polymyositis or dermatomyositis: a three-dimensional speckle-tracking echocardiography study. Int J Cardiovasc Imaging 34(5):683–693. 10.1007/s10554-017-1278-9 29168054 10.1007/s10554-017-1278-9

[20] Ballo P, Chiodi L, Cameli M et al (2012) Dilated cardiomyopathy and inclusion body myositis. Neurol Sci 33(2):367–370. 10.1007/s10072-011-0766-2 21922313 10.1007/s10072-011-0766-2

[21] Prutkin JM, Patton KK (2009) Ventricular tachycardia in a patient with inclusion-body myositis. Pacing Clin Electrophysiol 32(12):e36–39. 10.1111/j.1540-8159.2009.02534.x 19744271 10.1111/j.1540-8159.2009.02534.x

[22] Cox FM, Delgado V, Verschuuren JJ et al (2010) The heart in sporadic inclusion body myositis: a study in 51 patients. J Neurol 257(3):447–451. 10.1007/s00415-009-5350-9 19813068 10.1007/s00415-009-5350-9 PMC2837876

[23] Rosenbohm A, Buckert D, Kassubek J, Rottbauer W, Ludolph AC, Bernhardt P (2020) Sporadic inclusion body myositis: no specific cardiac involvement in cardiac magnetic resonance tomography. J Neurol 267(5):1407–1413. 10.1007/s00415-020-09724-4 31997038 10.1007/s00415-020-09724-4 PMC7184047

[24] Umair M, Asatryan B, Aliyari Ghasabeh M et al (2024) The specificity of Left Ventricular Bite-Like Fibrofatty replacement for diagnosis of Arrhythmogenic Right Ventricular Cardiomyopathy. JACC Cardiovasc Imaging 17(9):1113–1115. 10.1016/j.jcmg.2024.03.011 38727643 10.1016/j.jcmg.2024.03.011

[25] Peng A, Koffman BM, Malley JD, Dalakas MC (2000) Disease progression in sporadic inclusion body myositis: observations in 78 patients. Neurology 55(2):296–298. 10.1212/wnl.55.2.296 10908910 10.1212/wnl.55.2.296

